# Towards Cleaner Cities: Estimating Vehicle-Induced PM_2.5_ with Hybrid EBM-CMA-ES Modeling

**DOI:** 10.3390/toxics12110827

**Published:** 2024-11-19

**Authors:** Saleh Alotaibi, Hamad Almujibah, Khalaf Alla Adam Mohamed, Adil A. M. Elhassan, Badr T. Alsulami, Abdullah Alsaluli, Afaq Khattak

**Affiliations:** 1 Civil and Environmental Engineering Department, Faculty of Engineering—Rabigh Branch, King Abdulaziz University, Jeddah 21589, Saudi Arabia; 2 Department of Civil Engineering, College of Engineering, Taif University, Taif 21944, Saudi Arabia; hmujibah@tu.edu.sa (H.A.); aahassan@tu.edu.sa (A.A.M.E.); amalsaluli@tu.edu.sa (A.A.); 3 Department of Civil Engineering, College of Engineering, Bisha University, Bisha 61361, Saudi Arabia; kaamohamed@ub.edu.sa; 4 Department of Civil Engineering, College of Engineering and Architecture, Umm Al-Qura University, Makkah 24382, Saudi Arabia; btsulami@uqu.edu.sa; 5 Department of Civil, Structural and Environmental Engineering, Trinity College Dublin, D02 PN40 Dublin, Ireland

**Keywords:** air quality, PM_2.5_, explainable boosting machine, covariance matrix adaptation evolution strategy

## Abstract

In developing countries, vehicle emissions are a major source of atmospheric pollution, worsened by aging vehicle fleets and less stringent emissions regulations. This results in elevated levels of particulate matter, contributing to the degradation of urban air quality and increasing concerns over the broader effects of atmospheric emissions on human health. This study proposes a Hybrid Explainable Boosting Machine (EBM) framework, optimized using the Covariance Matrix Adaptation Evolution Strategy (CMA-ES), to predict vehicle-related PM_2.5_ concentrations and analyze contributing factors. Air quality data were collected from Open-Seneca sensors installed along the Nairobi Expressway, alongside meteorological and traffic data. The CMA-ES-tuned EBM model achieved a Mean Absolute Error (MAE) of 2.033 and an R^2^ of 0.843, outperforming other models. A key strength of the EBM is its interpretability, revealing that the location was the most critical factor influencing PM_2.5_ concentrations, followed by humidity and temperature. Elevated PM_2.5_ levels were observed near the Westlands roundabout, and medium to high humidity correlated with higher PM_2.5_ levels. Furthermore, the interaction between humidity and traffic volume played a significant role in determining PM_2.5_ concentrations. By combining CMA-ES for hyperparameter optimization and EBM for prediction and interpretation, this study provides both high predictive accuracy and valuable insights into the environmental drivers of urban air pollution, providing practical guidance for air quality management.

## 1. Introduction

Air pollution has become a pressing issue for both environmental sustainability and and public health in developing nations, intensifying in recent decades due to rapid industrial growth and urbanization [1,2]. Estimates show that nearly 7 million deaths each year can be traced to exposure to fine particulate matter with a diameter of less than 2.5 μm (PM_2.5_). Moreover, approximately 91% of the world’s population lives in areas where PM_2.5_ concentrations surpass the acceptable limits of 10–20 μg/m^3^ [3,4]. Both toxicological and epidemiological studies have established a strong association between PM_2.5_ exposure and heightened risks of cardiovascular and respiratory ailments, alongside an increased incidence of premature mortality linked to prolonged exposure [5,6,7,8]. The Global Burden of Disease (GBD) study ranks PM_2.5_ as the fifth leading risk factor for global mortality, accounting for approximately 4.2 million premature deaths annually [9].

Many rapidly growing nations, such as Saudi Arabia, India, and China, face significant challenges in managing air quality [10]. With high levels of transportation-related pollution, industrial emissions, and energy use, these countries are taking steps to combat rising air pollution as part of broader sustainability initiatives. Urban centers like Riyadh, Delhi, and Beijing see elevated PM_2.5_ levels due to population growth and a heavy reliance on gasoline-powered vehicles. To address these challenges, governments are promoting electric vehicles (EVs) and improving public transportation infrastructure. For example, Saudi Arabia has partnered with Lucid Motors to produce EVs domestically [11], while India and China are expanding charging infrastructure and offering EV subsidies. Investment in public transit, like Saudi Arabia’s Riyadh Metro and metro expansions in India and China, aims to reduce vehicle emissions by decreasing private car reliance. These initiatives are expected to significantly lower air pollutants such as PM_2.5_ and NO_x_, contributing to healthier urban environments.

Rapid development across many African countries has led to significant urbanization and a sharp rise in vehicle usage, thereby intensifying energy demand [12]. This growth has profoundly impacted air quality, particularly concerning PM_2.5_ levels resulting from vehicular emissions. Meteorological conditions are pivotal in intensifying PM_2.5_ concentrations, as they influence the dispersion, dilution, and deposition of these fine particles [13,14,15]. Research indicates that unfavorable meteorological conditions can lead to elevated PM_2.5_ levels, even when emissions are reduced, compared with scenarios with more favorable weather and higher emissions. Key factors such as fluctuations in humidity, wind speed, atmospheric pressure, and temperature play a critical role in shaping the spatial and temporal distribution of PM_2.5_ [16,17]. Furthermore, earlier studies have demonstrated that vehicular emissions are a major contributor to PM_2.5_ pollution in the environment. Specifically, vehicle-related PM_2.5_ accounts for 39.8% of the total PM_2.5_ concentration in Shanghai, 16% in New York, and 26% in Beijing [18]. However, there is a scarcity of research on vehicle-related PM_2.5_ emissions in developing countries. Therefore, this study seeks to estimate the concentration of PM_2.5_ emissions from vehicles and assess the impact of various traffic-related and environmental conditions on these emissions. We propose a new approach using the Explainable Boosting Machine (EBM) framework [19], with hyperparameter fine-tuning achieved via the Covariance Matrix Adaptation Evolution Strategy (CMA-ES) [20]. This is motivated by the following objectives:

- The EBM model was selected due to its strong predictive capabilities in forecasting PM_2.5._ Unlike black-box models, EBM maintains transparency and offers inherent interpretability, allowing stakeholders to understand the contributing factors [21,22,23,24].
- EBM is a Generalized Additive Model (GAM) that provides high interpretability by modeling feature effects independently. This aspect is crucial when assessing environmental risks such as PM_2.5_, as it enables clear identification of how variables like location, humidity, and temperature contribute to PM_2.5_ levels, providing actionable insights for policymakers and planners.
- To ensure optimal performance, the hyperparameters of EBM are fine-tuned using CMA-ES. CMA-ES is a robust evolutionary optimization algorithm known for efficiently navigating complex, high-dimensional search spaces [25]. Compared with traditional methods such as Grid Search or Random Search [26], CMA-ES is more adaptive and capable of handling non-linearities and interactions in the model, ensuring that EBM achieves its best possible performance on the PM_2.5_ dataset.

**Figure 1:**
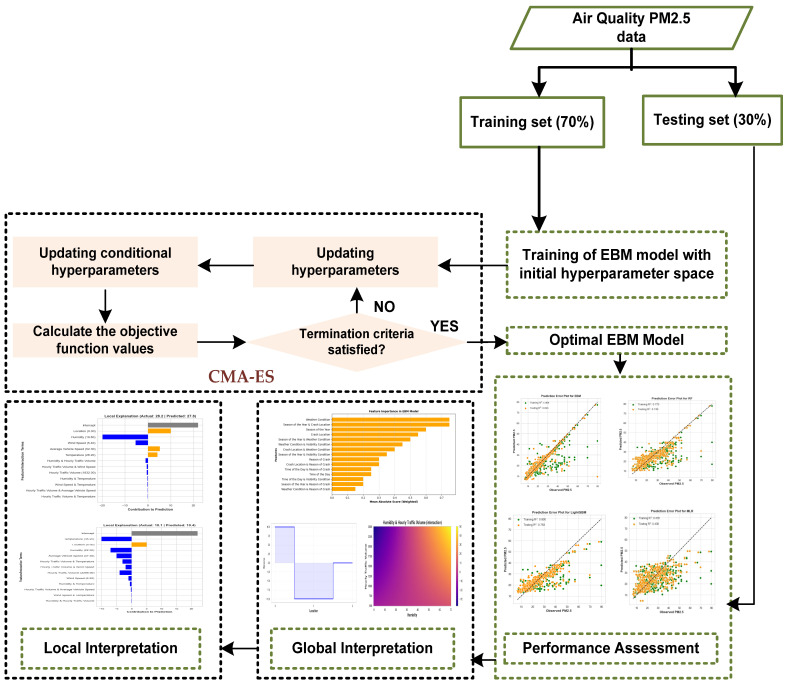
Proposed EBM-CMA-ES framework for the prediction and assessment of PM_2.5_.

This EBM-CMA-ES framework not only improves predictive accuracy but also preserves model transparency, making it well-suited for forecasting PM_2.5_. It empowers both prediction and interpretability, ensuring a comprehensive understanding of PM_2.5_ levels and their contributing factors. Figure 1 illustrates the proposed EBM-CMA-ES framework. The structure of this paper is organized as follows: Section 2 reviews the existing literature on statistical and machine learning models used for predicting PM_2.5_ levels, focusing on their strengths and limitations. Section 3 provides a detailed description of the study location and outlines the theoretical background of the methods employed, including the EBM framework and the CMA-ES for hyperparameter optimization. Section 4 evaluates and explains the model performance, conducts uncertainty analysis, and interprets the results with a focus on the inherent interpretability of EBM. Finally, Section 5 presents the conclusions and provides recommendations for future research and practical applications.

## 2. Related Work

PM_2.5_ has emerged as a significant public health concern due to its adverse effects on human health [27,28]. Extensive research highlights the crucial role of environmental and atmospheric factors, such as temperature, humidity, and wind patterns, in shaping ambient PM_2.5_ concentrations. To predict PM_2.5_ levels, various statistical models have been employed, drawing on these meteorological variables. A notable example is a multiple linear regression (MLR) model designed to estimate daily PM_2.5_ concentrations across different monitoring stations in the western United States. This model incorporates factors like prior day PM_2.5_ levels, fire radiative power, and aerosol optical depth from satellite data, providing an effective tool for forecasting PM_2.5_ fluctuations in response to changing environmental conditions [29]. Another study utilized a Bayesian ensemble approach to create a method that integrates aerosol optical depth (AOD) data from satellites with chemical transport model (CTM) simulations, thereby improving PM_2.5_ estimation [30]. Another study also applied MLR to forecast PM_2.5_ levels by utilizing various risk factors, including maximum and minimum noise, temperature, and humidity [31]. Statistical models, particularly MLR, are widely utilized due to their interpretability and straightforwardness. However, they possess significant drawbacks. Traditional statistical models are based on stringent assumptions, such as linearity, normality, homoscedasticity, and independence of residuals. Violation of these assumptions can result in biased or inaccurate outcomes. Additionally, these models often struggle with complex nonlinear relationships and interactions that frequently occur in real-world datasets. This limitation arises from their inherent structure, which is constrained by predefined assumptions and lacks the flexibility needed to adapt to the multidimensional characteristics of practical data scenarios where variables may not conform neatly to theoretical expectations. As a result, when data exhibit intricate behaviors or interdependencies, conventional approaches may fail to yield reliable or robust insights. Such limitations hinder their effectiveness in capturing complex patterns compared with more adaptable methodologies. Furthermore, statistical models may encounter challenges with large datasets or high-dimensional data due to computational inefficiencies or risks of overfitting, particularly when not managed with precision [32,33].

In contrast, Artificial Intelligence (AI) and, in particular, machine learning models, is increasingly preferred for several reasons. Machine learning models are adept at managing nonlinear and complex relationships more effectively than traditional statistical approaches [34]. They can automatically detect interactions between variables without explicit specification. The machine learning models are designed to handle large-scale data and can be easily automated to learn from new data continuously, improving their predictions over time [35]. Researchers around the world are increasingly turning to machine learning models to predict PM_2.5_ concentrations. For instance, a study conducted in northern Taiwan made use of the self-organizing map (SOM) technique. This approach clusters high-dimensional data into a comprehensible two-dimensional topological map, effectively highlighting the spatial and temporal distribution of PM_2.5_ levels. This method enhanced the visualization and understanding of how PM_2.5_ concentrations fluctuate over different locations and periods [36]. Another study employed a Random Forest (RF) model to estimate the PM_2.5_ levels across China from 2005 to 2016. The proposed model significantly outperformed traditional statistical regression models in capturing spatial variability and reducing prediction errors at daily, monthly, and annual time scales [37]. Another similar study conducted in various regions of China employed an ensemble machine learning approach that combines Random Forest (RF), generalized additive models, and extreme Gradient Boosting (XGBoost) and demonstrated a strong PM_2.5_ prediction accuracy [38]. In order to consider both machine learning and deep learning models, a study conducted in the Hunan province of China utilized XGBoost and a fully connected neural network (FCNN) to predict PM_2.5_ concentrations using data from meteorological parameters and PM_2.5_ measurements. It was observed that the XGBoost model outperformed the neural network in predicting PM_2.5_ [39]. A study conducted in Malaysia employed RF and Support Vector Machine (SVM) to estimate PM_2.5_ concentrations by combining satellite data, ground-measured pollutants, and meteorological factors. The RF model performed better than SVM in predicting PM_2.5_ [40]. In addition, deep learning models have also been used to predict PM_2.5_ concentration. A study employed a weighted long short-term memory extended model (WLSTME) to improve PM_2.5_ prediction accuracy by considering site density and wind conditions. The WLSTME integrated neighbor site data, historical PM_2.5_ concentrations, and meteorological data, outperforming previous methods [41]. Similarly, some other researchers employed deep convolutional neural networks (CNNs) to estimate PM_2.5_ levels. This research involved generating a hallucinated reference image, computing discrepancy maps, and predicting PM_2.5_ concentrations using extracted features [42]. Another study employed deep learning-based recurrent neural networks (RNNs), including Long Short-Term Memory (LSTM), Bi-LSTM (Bidirectional LSTM), and Bidirectional Gated Recurrent Unit (Bi-GRU) models, alongside a CNN to predict PM_2.5_ concentration using meteorological data from 2017 to 2019 from Taiwan [43].

To the best of our knowledge, no researcher has previously utilized the EBM in combination with the CMA-ES for predicting PM_2.5_ concentrations. Therefore, this study adopts the EBM model to take advantage of its inherent interpretability and predictive capabilities. By employing CMA-ES for hyperparameter tuning, the EBM model performance is further enhanced, allowing it to achieve higher accuracy in PM_2.5_ prediction. In addition, the inherent transparency of the EBM framework ensures that the model not only delivers accurate predictions but also provides clear, interpretable insights into the influence of various input features on PM_2.5_ levels, providing a deeper understanding of the contributing risk factors.

## 3. Materials and Methods

### 3.1. Study Location and Data

**Figure 2:**
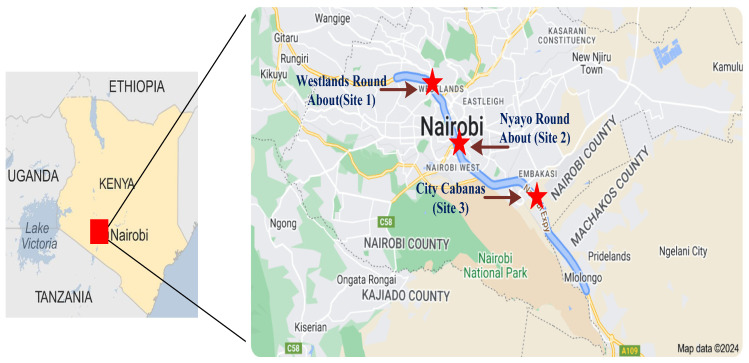
Sites for the data collection along Nairobi expressway.

The Nairobi Expressway serves as a critical transportation corridor, connecting Nairobi’s urban center to Jomo Kenyatta International Airport (JKIA). This 27 km (17 miles) six-lane dual carriageway runs along the central reservations of Mombasa Road, beginning at Mlolongo, extending through Uhuru Highway, and concluding at James Gichuru Road, as illustrated in Figure 2. It is a vital route for commuters, particularly those traveling to and from the airport.

For this study, data collection was conducted at three strategically chosen locations along the Nairobi Expressway corridor. Monitoring occurred for 12 h per day over seven consecutive days, focusing on peak hours when traffic flow and related emissions are most significant. To capture potential seasonal variations in PM_2.5_ concentrations, data were collected during three distinct periods: 23–29 August 2021 (representing the dry season), 13–18 December 2021 (peak holiday season), and 21–27 March 2022 (post-holiday, also dry). August represents the dry season in Kenya, where PM_2.5_ levels may be elevated due to reduced rainfall and increased dust resuspension. December coincides with the peak holiday season, likely increasing traffic volumes and vehicle-related emissions. March, as a dry month post holiday, allows for observations of typical daily traffic patterns and ambient air quality outside peak travel periods. Table 1 provides detailed descriptions of these monitoring sites.

Traffic volumes were systematically recorded across various vehicle categories, including motorcycles, passenger vehicles, buses, and goods vehicles of differing capacities (light, medium, heavy, and articulated trucks). The comprehensive datasets were compiled on ambient air pollutant concentrations using calibrated Open-Seneca sensors. The sensors were calibrated before deployment by cross-referencing with a reference-grade air quality monitoring station, involving both laboratory testing to establish baseline accuracy and field calibration to account for environmental variables such as temperature and humidity. This calibration process ensured that PM_2.5_ measurements were accurate and consistent across the three monitoring locations. Along with pollutant data, hourly traffic volume, as well as average vehicle speeds and meteorological parameters, including humidity, wind speed, and temperature, were recorded to provide a holistic view of factors influencing air quality along the Nairobi Expressway.

### 3.2. Hybrid EBM-CMA-ES Framework

#### 3.2.1. Theoretical Overview of EBM

The EBM is an advanced machine learning model designed to balance high predictive accuracy with interpretability. It combines the principles of the Generalized Additive Model (GAM) and boosting algorithms to create a model that is both powerful and interpretable. This makes EBM particularly useful in domains where understanding the rationale behind predictions is crucial. The GAM represents the outcome as an additive function of the predictors, allowing for straightforward interpretation. For any $m^{th}$ data point within a data, the general form of a GAM is given in Equation (1).

(1) $$y=\varphi_{0}+\Sigma \varphi_{m}\left(x_{m}\right)+\varepsilon$$

where the following hold:

- y is the predicted outcome.
- $\varphi_{0}$ is the intercept.
- $\varphi_{m}\left(x_{m}\right)$ are the shape functions for each feature $x_{m}$.
- ε is the error term.

Each shape function $\varphi_{m}\left(x_{m}\right)$ captures the relationship between the feature $x_{m}$ and the outcome y. This allows us to visualize and understand the effect of individual features on the prediction. The boosting is an ensemble technique that enhances model performance by combining multiple weak learners. The process involves training weak learners sequentially, where each new learner focuses on correcting the errors of the previous ones. The objective is to minimize a loss function, typically using gradient descent. The process EBM modeling begins with an initial prediction, often the mean of the target variable as given by Equation (2)

(2) $${\hat{y}}^{\left(0\right)}=\bar{y}$$

where the following hold:

- ${\hat{y}}^{\left(0\right)}$ is the initial prediction.
- $\bar{y}$ is the mean of the target variable.

The model iteratively refines the predictions by learning shape functions for each individual feature. Each iteration t involves computing residuals, fitting weak learners, updating shape function, and updating predictions. The residuals are calculated as the difference between the actual target values and the current predictions as shown in Equation (3).

(3) $$r^{\left(t\right)}=y-{\hat{y}}^{\left(t-1\right)}$$

where the following hold:

- $r^{\left(t\right)}$ is the residual at iteration t.
- y is the actual target value.
- ${\hat{y}}^{\left(t-1\right)}$ is the prediction from the previous iteration.

For each feature $x_{i}$, fit a weak learner (e.g., a decision tree) to the residuals. This step focuses on learning the shape function that $\varphi_{m}\left(x_{m}\right)$ best explains the residuals for that feature as given by Equation (4).

(4) $$\varphi_{m}^{\left(t\right)}\left(x_{m}\right)\leftarrow \mathrm{FitWeakLearner}\left(r^{\left(t\right)},x_{m}\right)$$

It is then followed by updating the shape functions for each individual feature by adding the contribution from the current iteration, scaled by a learning rate η as given by Equation (5).

(5) $$\varphi_{m}\left(x_{m}\right)\leftarrow \varphi_{m}\left(x_{m}\right)+\eta \cdot \varphi_{m}^{\left(t\right)}\left(x_{m}\right)$$

where the following hold:

- $\varphi_{m}\left(x_{m}\right)$ is the updated shape function for feature $x_{m}$.
- η is the learning rate, controlling the step size of the update.

It also includes two-dimensional interactions between the features. The two-dimensional interactions can be rendered as heat-maps on a two-dimensional plane, the model that includes two-dimensional interaction is also interpretable. Thus, the overall prediction may be updated by adding the contributions from the updated shape functions and two-dimensional interaction, as given by Equation (6).

(6) $${\hat{y}}^{\left(t\right)}={\hat{y}}^{\left(t-1\right)}+\sum_{m=`}^{\left(M\right)}f_{m}^{\left(t\right)}(x_{m})+\sum_{m=1}^{\left(M\right)}\sum_{n=1`}^{\left(N\right)}f_{m,n}^{\left(t\right)}(x_{m},x_{n})$$

where the following hold:

- ${\hat{y}}^{\left(t\right)}$ is the updated prediction at iteration t.
- $\sum_{m=`}^{\left(M\right)}f_{m}^{\left(t\right)}(x_{m})$ captures the contributions from each individual feature.
- $\sum_{m=1}^{\left(M\right)}\sum_{n=1`}^{\left(N\right)}f_{m,n}^{\left(t\right)}(x_{m},x_{n})$ captures the contributions from interactions between pairs of features.

The iterative process continues until a stopping criterion is met. Common stopping criteria include (1) maximum number of iterations T; (2) convergence of the loss function, i.e., when changes in the loss function fall below a certain threshold. The iteration process stops when t≥T or ΔLoss<ε, where t≥T is the maximum number of iteration, and ε is the threshold for convergence.

#### 3.2.2. Interpretation of EBM

One of the key interpretability features of EBM is the shape functions $\varphi_{m}\left(x_{m}\right)$. Each shape function represents the relationship between a feature and the target variable. These functions can be visualized to understand how changes in a feature affect the prediction. For instance, if $\varphi_{m}\left(x_{m}\right)$ is a straight line, it indicates a linear relationship between $x_{m}$ and y. In the case where $\varphi_{m}\left(x_{m}\right)$ is a curve, it indicates a nonlinear relationship, capturing more complex interactions between $x_{m}$ and y. The additive nature of EBM allows for a clear interpretation of each feature’s contribution to the final prediction. By examining the shape functions, one can see how each feature influences the outcome. A positive slope in $\varphi_{m}\left(x_{m}\right)$ indicates that higher values of $x_{m}$ lead to higher predicted values of y. A negative slope in $\varphi_{m}\left(x_{m}\right)$ indicates that higher values of $x_{m}$ lead to lower predicted values of y. The pairwise interaction involve heatmaps or contour plots, where $x_{m}$ and $x_{n}$ are on the axes, and the calculated $f(x_{m},x_{n})$ values fill the plot. This visualization helps in understanding which combinations of m and n contribute most to the outcome.

#### 3.2.3. Covariance Matrix Adaptation Evolution Strategy (CMA-ES)

CMA-ES is an advanced evolutionary algorithm specifically developed for optimization in continuous parameter spaces. It is part of the evolution strategies family, inspired by the principles of natural evolution. It adapts the covariance matrix of the search distribution to effectively explore the search space, concentrating on the most promising regions. The algorithm iteratively updates a population of candidate solutions, utilizing a multivariate normal distribution whose mean and covariance matrix are dynamically adjusted based on the performance of the selected solutions.

The core principle of CMA-ES is to represent the search distribution as a multivariate Gaussian, with its mean and covariance matrix being iteratively refined. It starts with an initial mean vector $m_{o}$ and covariance matrix $C_{o}$ and set initial step-size $\sigma_{o}$ and population size $\lambda_{o}$. Generate offspring by sampling from a multivariate normal distribution as given by Equation (7)

(7) $$x_{k}\sim N\left(m_{t},\sigma_{t}^{2}C_{t}\right)$$

where $m_{t}$ is the mean vector, $\sigma_{t}$ is the step-size, and $C_{t}$ is the covariance matrix at iteration t. Evaluate the objective function $f(x_{k})$ for each offspring $x_{k}$. Select the top μ solutions based on their fitness values to form a new mean as shown by Equation (8)

(8) $$m_{t+1}=\sum_{i=1}^{\mu }\omega_{i}x_{i}$$

where $\omega_{i}$ denotes the weights allocated to each selected solution. The covariance matrix $C_{t+1}$ is updated and step-sizes $\sigma_{t+1}$ are represented by Equations (9) and (10), respectively.

(9) $$C_{t+1}=\left(1-c_{c}\right)C_{t}+c_{c}\sum_{i=1}^{\mu }\omega_{i}\left(x_{i}-m_{t}\right){\left(x_{i}-m_{t}\right)}^{T}$$

(10) $$\sigma_{t+1}=\sigma_{t}\exp\left(\frac{c_{\sigma }}{d_{\sigma }}\left(\frac{∥p_{c,t+1}∥}{E∥Ν\left(0,1\right)∥}-1\right)\right)$$

where $c_{c}$, $c_{\sigma }$, and $d_{\sigma }$ represent the learning rates, and $p_{c,t+1}$ denote the evolution path. Continue the process until the convergence criteria are met, such as attaining the maximum number of iterations or achieving the desired fitness level.

For the EBM model, the key hyperparameters requiring careful optimization include the learning rate, the maximum number of bins, the maximum number of interaction bins, and the number of boosting iterations [44]. The learning rate determines the step size at each boosting iteration. A lower learning rate allows the model to learn more gradually, reducing the risk of overfitting and often improving accuracy, while a higher learning rate speeds up training but may compromise EBM model accuracy. The maximum number of bins defines the number of discrete bins used to partition continuous features. A higher number of bins allows for more detailed feature representation but increases EBM model complexity. This parameter affects how well the model captures variable interactions and feature effects. The maximum number of interaction bins controls the number of bins used specifically for pairwise feature interactions, allowing the model to capture important dependencies between features. Optimizing this parameter enhances the EBM model’s ability to capture complex feature relationships without overcomplicating the structure. The number of boosting iterations refers to the total number of boosting rounds. Too few iterations can lead to underfitting, as the model may not fully capture data patterns, while too many can cause overfitting by making the model overly complex.

### 3.3. Competitive Machine Learning Models

In addition to EBM, several other machine learning models were used to analyze PM_2.5_ concentration, including XGBoost, RF, LightGBM, and AdaBoost. Each model has distinct strengths. XGBoost and LightGBM are both gradient-boosting techniques optimized for efficiency, while Random Forest is known for its robustness in various tasks. AdaBoost, on the other hand, is useful for improving weak learners through iterative weighting. Table 2 shows the summary table of different machine learning models.

### 3.4. Performance Measures

For evaluating the performance of regression models in machine learning, several metrics are commonly used. These include Mean Absolute Error (MAE), Root Mean Squared Error (RMSE), Mean Squared Error (MSE), and the coefficient of determination (*R*^2^). Each metric provides different insights into the accuracy and performance of a model. MAE quantifies the mean magnitude of prediction errors within a dataset, disregarding the directionality of these errors. Specifically, it computes the average across a test sample of the absolute variances between each predicted value and its corresponding actual observation, treating all individual variances uniformly, as given in Equation (11).

(11) $$MAE=\frac{1}{n}\sum_{i=1}^{n}\left|y_{i}-{\hat{y}}_{i}\right|$$

MSE calculates the average of the squared differences between estimated values and the actual values. This metric represents the mean square error across the dataset, as specified in Equation (12).

(12) $$MSE=\frac{1}{n}{\sum_{i=1}^{n}\left(y_{i}-{\hat{y}}_{i}\right)}^{2}$$

RMSE represents the square root of the average of squared errors, as shown in Equation (13). It serves as a measure of the accuracy with which the model predicts the response.

(13) $$RMSE=\sqrt{\frac{1}{n}{\sum_{i=1}^{n}\left(y_{i}-{\hat{y}}_{i}\right)}^{2}}$$

*R*^2^, also known as the coefficient of determination, measures the proportion of variance in the dependent variable that is predictable from the independent variables. It quantifies how closely data points align with the fitted regression line, as shown in Equation (14).

(14) $$R^{2}=1-\frac{{\sum_{i=1}^{n}\left(y_{i}-{\hat{y}}_{i}\right)}^{2}}{{\sum_{i=1}^{n}\left(y_{i}-{\bar{y}}_{i}\right)}^{2}}$$

where the following hold:

- *n* is the number of data points.
- $y_{i}$ is the actual value.
- ${\hat{y}}_{i}$ is the predicted value.
- ${\bar{y}}_{i}$ is the mean of the actual values y.

## 4. Results and Discussion

This study utilizes air quality data collected from sensors positioned along the Nairobi Expressway, supplemented by meteorological and traffic data, including humidity, hourly temperature, average traffic volume, average vehicle speed, wind speed, and site location. To handle missing values in the dataset, the K-Nearest Neighbors (KNNs) method was applied, imputing the missing data points based on the similarity of neighboring values [45]. This approach preserves the dataset’s overall quality, making it more robust for predictive modeling. The dataset was divided into two subsets: 70% of the data was allocated for training and validation, while the remaining 30% was reserved for testing. This split follows a widely accepted machine learning practice, enabling model development and hyperparameter tuning on the training–validation set, while the test set remains unseen for performance evaluation.

**Figure 3:**
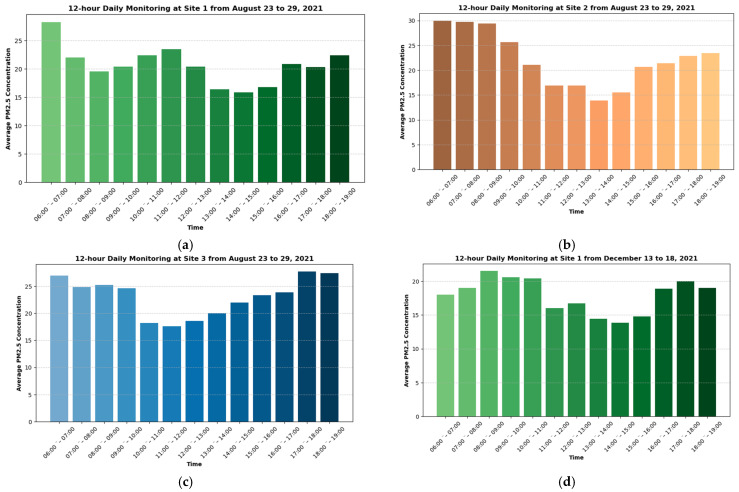

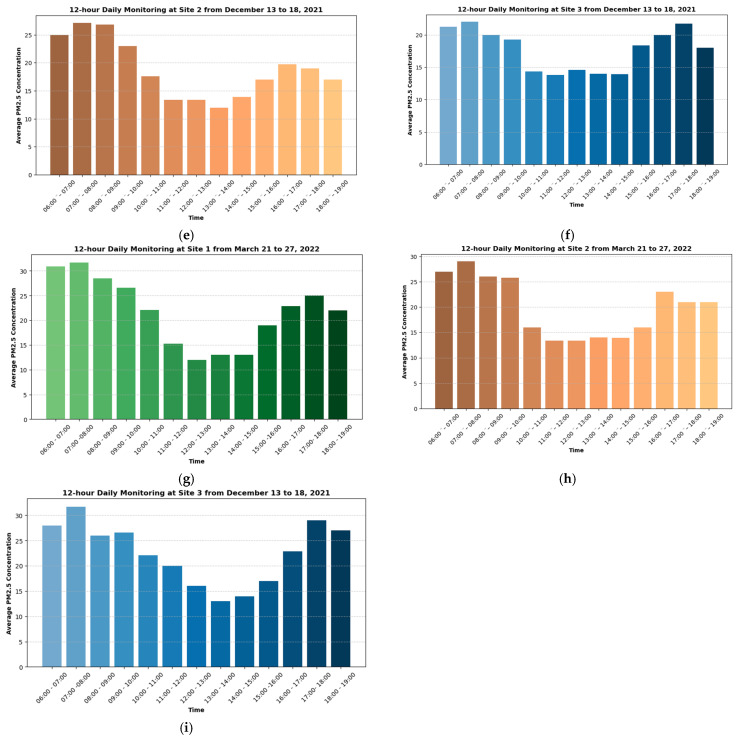
Twelve-hour daily variation in average PM_2.5_ at different sites along Nairobi expressway.

For the model development, the EBM was employed with hyperparameter optimization performed through the CMA-ES. The EBM’s inherent interpretability was a key factor in its selection, as it allows for transparent analysis of feature contributions to the predictions. The performance of the EBM model was evaluated on the test set and compared with several alternative machine learning models, including RF [46], XGBoost [47], LightGBM [48], AdaBoost [49], and the MLR [50]. Among the features used in this study, site location was treated as a categorical variable, where Westlands Roundabout (Site 1) was encoded as 0, Nyayo Roundabout (Site 2) as 1, and City Cabanas (Site 3) as 2. All models were implemented in Python 3.7.1, with Table 3 presenting the descriptive statistics for the input factors used in the analysis. As discussed previously, a 12 h daily monitoring was conducted for each of the three sites. Consequently, Figure 3 illustrates the average PM_2.5_ concentration at different times of the day.

### 4.1. Fine-Tuning of Hyperparameters via CMA-ES

To optimize the performance of the EBM model, hyperparameters were fine-tuned using the CMA-ES [51]. The primary objective of the tuning process was to maximize the *R*^2^ value, a metric that indicates the predictive performance of the model by measuring the proportion of variance explained by the model predictions for comparative purposes, and the hyperparameters of other models, including XGBoost, RF, LightGBM, and AdaBoost, were also optimized. The best hyperparameters identified for the EBM model and other comparative models are summarized in Table 4.

### 4.2. Prediction Results and Comparative Analysis

The comprehensive performance evaluation across multiple machine learning models, as shown in Table 5, presents the performance metrics for predicting PM_2.5_ concentrations. Among all models, the EBM, fine-tuned using the CMA-ES, demonstrated the most robust and accurate predictions. EBM outperformed the other models in both the training and testing datasets, with lower error rates and higher *R*^2^ values, highlighting its predictive superiority and robustness. The EBM-CMA-ES model achieved an MAE of 1.615 on the training set and 2.033 on the testing set, an MSE of 15.539 on the training set and 28.134 on the testing set, an RMSE of 3.942 on the training set and 5.304 on the testing set, and an *R*^2^ of 0.904 on the training set and 0.843 on the testing set. These results confirm that the EBM model not only performs well on the training data but also generalizes effectively to unseen data, making it highly suitable for PM_2.5_ concentration forecasting.

In comparison, the second-best-performing model was XGBoost, which delivered an MAE of 3.58 on the training set and 3.84 on the testing set, an MSE of 31.56 on the training set and 34.58 on the testing set, an RMSE of 5.62 on the training set and 5.88 on the testing set, and an *R*^2^ of 0.813 on the training set and 0.782 on the testing set. While XGBoost showed relatively strong performance, its error rates were higher, and its R^2^ values were lower than those of the EBM model, indicating that it was less effective at capturing the underlying patterns in the data. At the other end of the spectrum, the MLR model displayed the weakest performance. It yielded an MAE of 7.55 on the training set and 7.23 on the testing set, an MSE of 97.98 on the training set and 89.12 on the testing set, an RMSE of 9.95 on the training set and 9.44 on the testing set, and an *R*^2^ of 0.418 on the training set and 0.438 on the testing set. These results indicate that MLR struggled to capture the complex relationships within the dataset, making it the least suitable model for predicting PM_2.5_ concentrations.

**Figure 4:**
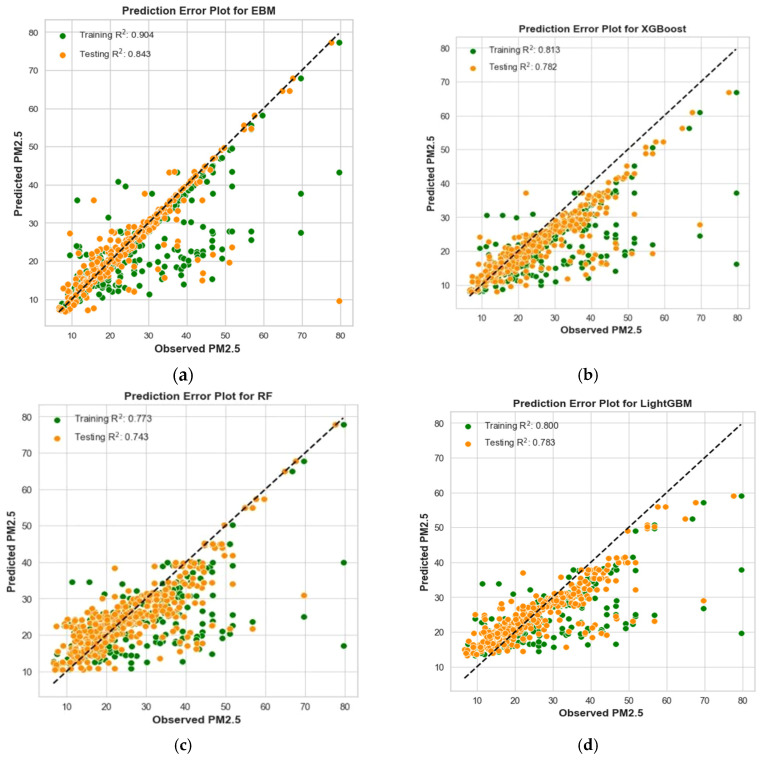

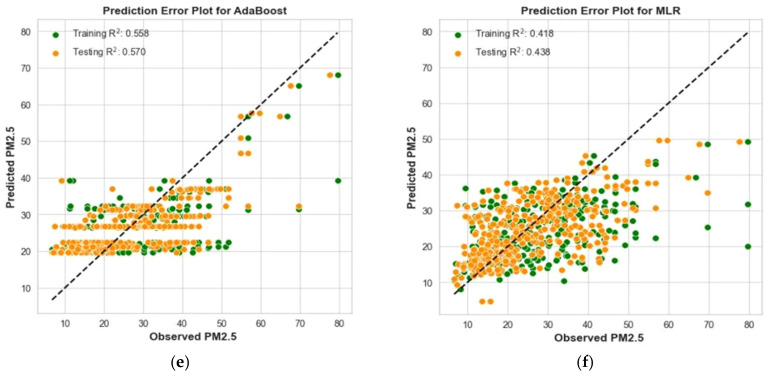
Prediction error plots using both training and testing datasets: (**a**) EBM; (**b**) XGBoost; (**c**) RF; (**d**) LightGBM; (**e**) AdaBoost; (**f**) MLR.

The prediction error plots in Figure 4 provide a clear visualization of the performance differences among the various models. These plots compare predicted values with actual data points, with a 45-degree reference line representing perfect predictions. The hybrid EBM-CMA-ES model stands out due to its close alignment with the reference line, both in the training and testing datasets. The dense clustering of data points along the line highlights the model’s high accuracy and low error, reinforcing its superior performance in predicting PM_2.5_ levels. In contrast, the prediction error plots for alternative models, including XGBoost, Random Forest, LightGBM, AdaBoost, and MLR, show greater dispersion of points around the 45-degree line. This scattering reflects their comparatively lower predictive accuracy, with more variability in their ability to match actual values. The broader distribution of data points away from the ideal line demonstrates that these models, while still effective to varying degrees, are less reliable than the EBM-CMA-ES model in accurately forecasting PM_2.5_ levels.

### 4.3. Uncertainty Analysis

**Figure 5:**
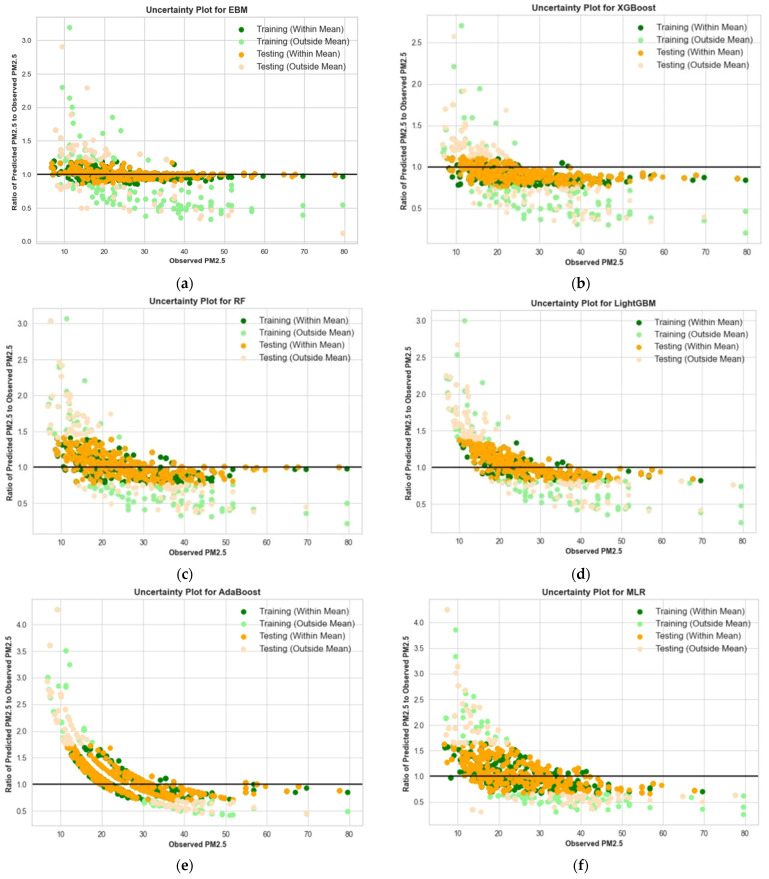
Uncertainty analysis of the machine learning model by plotting the ratio of predicted PM_2.5_ to the observed PM_2.5_ vs observed PM_2.5_: (**a**) EBM model (**b**) XGBoost model (**c**) RF model; (**d**) LightGBM model; (**e**) AdaBoost model; (**f**) MLR model.

The uncertainty analysis evaluates the variability and reliability of each machine learning model’s predictions by comparing the ratio of predicted PM_2.5_ concentrations to observed PM_2.5_ concentrations against the observed values. Figure 5a–f present these ratios for each model, while Table 6 summarizes the mean ratio and standard deviation for each model.

In Figure 5, the plot for each model provides a visual representation of how well the model’s predictions align with the observed PM_2.5_ values. For instance, Figure 5a shows that the EBM model has the most consistent performance, with the ratio of predicted to observed PM_2.5_ values clustering tightly around 1.0, indicating minimal deviation from the observed values. In contrast, Figure 5e,f, which represents the AdaBoost and MLR models, respectively, shows more scattered data points, suggesting higher variability in predictions. Table 6 further supports this observation by providing the mean ratio and standard deviation for each model’s predictions. The EBM model has a mean ratio close to 1.0024, indicating near-perfect accuracy and a relatively low standard deviation of 0.178, which confirms the model’s high precision and reliability. Similarly, the XGBoost and LightGBM models perform reasonably well, with mean ratios of 0.942 and 0.934, respectively, and moderate standard deviations, highlighting their stability. However, the AdaBoost and MLR models show considerably higher uncertainty, with mean ratios of 0.786 and 0.772, respectively, and the highest standard deviations (0.304 and 0.313). This indicates that these models have larger deviations in predictions, suggesting lower reliability and less accuracy in forecasting PM_2.5_ concentrations.

### 4.4. EBM Interpretation

In this section, we interpret our proposed EBM model, which emerged as the best-performing model for PM_2.5_ prediction. By employing the EBM model’s inherent interpretability, we are able to gain valuable insights into the factors that contribute to PM_2.5_ concentrations. We begin with a global interpretation by examining the feature importance plot and then proceed to local interpretations to explore how these factors affect individual predictions.

#### 4.4.1. EBM Global Interpretation

**Figure 6:**
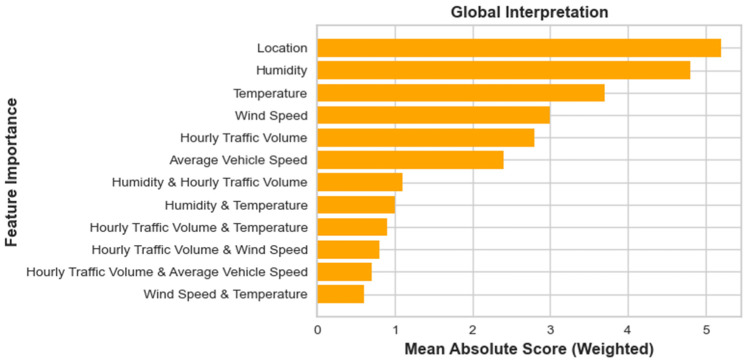
Global factors importance analysis via EBM.

The feature importance plot, as shown in Figure 6, generated from the EBM model, provides a ranked list of features based on their impact on the model’s predictions. The top three most significant individual factors contributing to PM_2.5_ concentration predictions are location, humidity, and temperature, each of which plays a distinct role in PM_2.5_ forecasting. Location stands out as the most critical factor in the model. This is likely because different locations along the Nairobi Expressway experience varying levels of pollution due to local sources such as traffic density, industrial activities, and proximity to urban centers. Location-specific factors like the presence of high-emission vehicles or specific meteorological conditions at certain sites could explain why this feature is highly influential. Humidity is the second most significant feature. Its importance likely stems from its ability to influence the behavior of airborne particles. High humidity can lead to the aggregation of particulate matter, increasing PM_2.5_ concentrations. Alternatively, during low humidity conditions, dry air can enhance the resuspension of particles from surfaces, further elevating PM_2.5_ levels. Therefore, fluctuations in humidity are strongly tied to changes in air pollution. Similarly, Temperature is the third most important individual feature. The relationship between temperature and PM_2.5_ levels could be attributed to several factors, including the impact of temperature on atmospheric mixing and chemical reactions. Warmer temperatures may reduce the stability of the atmosphere, allowing pollutants to disperse more easily, while cooler temperatures could trap pollutants closer to the ground. Furthermore, temperature can affect the formation of secondary particles, which contribute to PM_2.5_ levels.

In addition, the most significant interaction term is between humidity and hourly traffic volume, indicating that the combined effect of these two features plays an important role in PM_2.5_ concentration levels. This interaction likely highlights the dual impact of traffic emissions and atmospheric conditions. Increased traffic volume results in higher emissions of particulate matter, particularly from combustion engines. When traffic levels rise, PM_2.5_ concentrations are expected to increase. The interaction with humidity suggests that the effect of traffic on PM_2.5_ concentrations may vary depending on the moisture content in the air. Under high humidity conditions, the particles emitted by vehicles could cluster more easily, resulting in higher recorded PM_2.5_ levels. Conversely, at lower humidity levels, the particles might behave differently, potentially reducing their clustering but increasing their dispersal.

**Figure 7:**
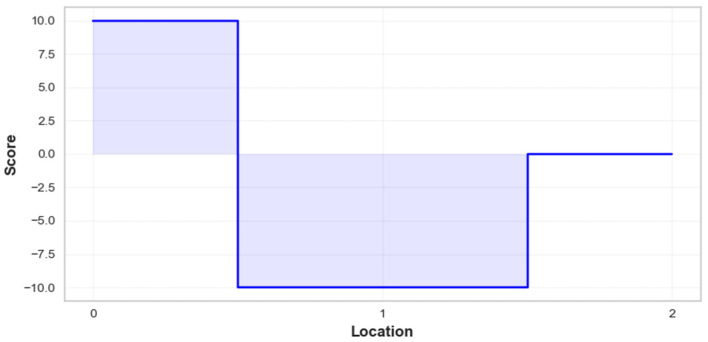
Influence of location on PM_2.5_ concentrations.

Figure 7 illustrates the impact of three key locations, including Westlands Roundabout (Location 1), Nyayo Roundabout (Location 2), and City Cabanas (Location 3), on PM_2.5_ concentrations. At the Westlands Roundabout, the score is strongly positive, around 10, indicating that this location contributes significantly to higher PM_2.5_ levels, likely due to heavy traffic and congestion. In contrast, Nyayo Roundabout shows a sharp drop in the score to around −10, reflecting a negative impact on PM_2.5_ levels, possibly due to smoother traffic flow or more favorable environmental conditions, which help reduce pollution. Lastly, City Cabanas has a score close to 0, suggesting a neutral impact on PM_2.5_ concentrations, with minimal influence on pollution levels. The filled blue areas highlight the magnitude of these effects, with Westlands being a significant contributor to pollution, Nyayo acting as a reducer, and City Cabanas having a negligible impact.

**Figure 8:**
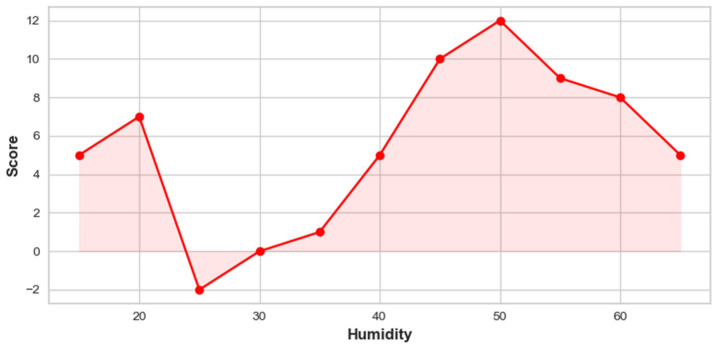
Influence of humidity on PM_2.5_ concentrations.

Similarly, Figure 8 illustrates the relationship between humidity and its impact on PM_2.5_ concentrations. As humidity increases from 15 to 65, its influence on PM_2.5_ levels fluctuates in a nonlinear pattern. Initially, at lower humidity levels around 15 to 25, the score shows a slight positive contribution, indicating a modest effect on PM_2.5_ concentrations. As humidity rises to around 30, the score dips briefly, showing that moderate humidity has a reducing effect, leading to a temporary decrease in concentrations. However, as humidity continues to increase beyond 35, the score rises significantly, indicating that higher humidity levels have a more pronounced positive impact on PM_2.5_ concentrations. This illustrates that when humidity reaches around 40 to 55, the concentration of PM_2.5_ particles increases considerably. Mechanisms such as particle deliquescence and aqueous-phase chemical reactions play their roles at elevated humidity. Deliquescence occurs when hygroscopic particles absorb moisture at specific humidity thresholds, transitioning from solid to aqueous phases, which enhances their ability to act as reaction sites for atmospheric chemicals. This process facilitates the formation of secondary inorganic aerosols, notably ammonium nitrate, under humid conditions. Research indicates that ammonium nitrate formation is more efficient in moist environments due to the increased solubility and reactivity of precursor gases like ammonia and nitric acid in aqueous aerosols [52]. Furthermore, studies have shown that the deliquescence and efflorescence relative humidities of aerosol particles are critical in determining their phase states and subsequent chemical reactivity, directly impacting PM_2.5_ levels [53]. The score peaks at the highest humidity levels, around 55 to 60, although the upward trend diminishes slightly towards the end, implying that while very high humidity still increases PM_2.5_ levels, the effect stabilizes.

**Figure 9:**
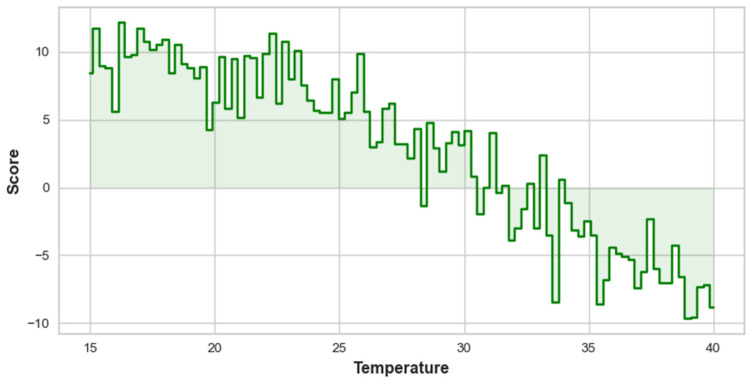
Influence of temperature on PM_2.5_ concentrations.

Figure 9 illustrates the relationship between temperature and its impact on PM_2.5_ concentrations. As the temperature increases from 15 to 40, the score shows a clear downward trend. Initially, at lower temperatures, particularly between 15 and 20, the score rises significantly, indicating a strong positive influence on PM_2.5_ concentrations. However, as temperatures exceed 20, the score begins to decline and eventually drops below 0 around 30, suggesting that higher temperatures have a diminishing effect, leading to no impact on PM_2.5_ levels. This trend implies that as temperatures rise, the environment’s capacity to maintain or increase PM_2.5_ concentrations decreases. The shaded area under the curve highlights the range of scores corresponding to specific temperature intervals, with the light green shading emphasizing the transition from a positive influence to a neutral or negative effect. Overall, the plot indicates that while lower temperatures contribute significantly to PM_2.5_ concentrations, higher temperatures are associated with a reduction in influence, ultimately leading to negligible effects on pollution levels.

**Figure 10:**
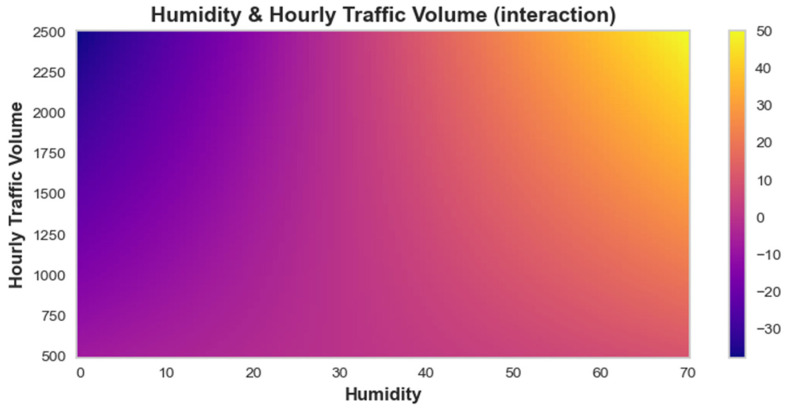
EBM-based heatmap for the interaction of humidity and hourly traffic volume.

In the case of feature interaction, the EBM heatmap illustrates the interaction between humidity and hourly traffic volume, with a color gradient indicating the corresponding interaction scores, as shown in Figure 10. Areas with humidity levels below 40 are predominantly shaded in purple, illustrating a minimal effect on PM_2.5_ concentrations. In contrast, as humidity increases to between 40 and 60, the colors transition to yellow and orange, indicating a strong positive influence on PM_2.5_ levels, particularly when combined with high traffic volumes (above 1500). This shows that optimal conditions for higher PM_2.5_ concentrations occur within this humidity and traffic volume range. The heatmap effectively communicates that higher humidity and traffic volumes can exacerbate air pollution, making it essential to monitor these parameters for effective environmental management.

#### 4.4.2. EBM Local Interpretation

**Figure 11:**
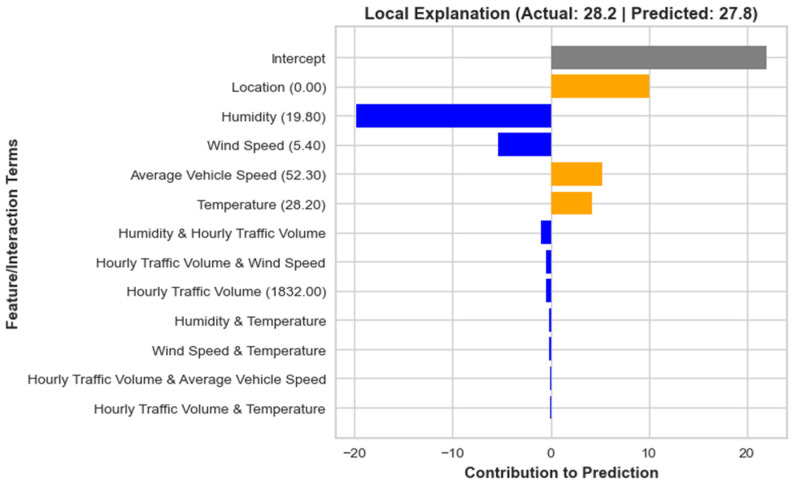
EBM-based local interpretation of Sample # 12 in testing dataset.

In addition to providing a global interpretation of the overall model’s behavior, the EBM can also be used for local interpretation, offering insights into how individual features influence the prediction for specific samples. In this study, we consider two randomly selected samples, i.e., sample #12 and sample #38 of the testing dataset. For sample #12, as shown in Figure 11, the model reveals the contributions of various factors to the predicted outcome of 27.8, allowing us to understand the specific impact of the most important features in this case. The feature with the strongest positive contribution is location (0.00), which adds 10 units to the prediction. This indicates that the sample’s location is a key driver in increasing the predicted value.

On the other hand, humidity (19.80) has a significant negative contribution of approximately −19.8 units, showing that the humidity levels, in this case, strongly reduce the prediction. Finally, wind speed (5.40) also contributes negatively, lowering the predicted value by −5.4 units, showing that higher wind speeds are associated with a reduction in the outcome for this sample. In this local interpretation, we see that while location plays a major positive role, humidity and wind speed act as substantial negative influences, shaping the final prediction together. This level of insight helps to explain why the model predicted 27.8 for this particular sample, providing transparency into the model’s decision-making process.

**Figure 12:**
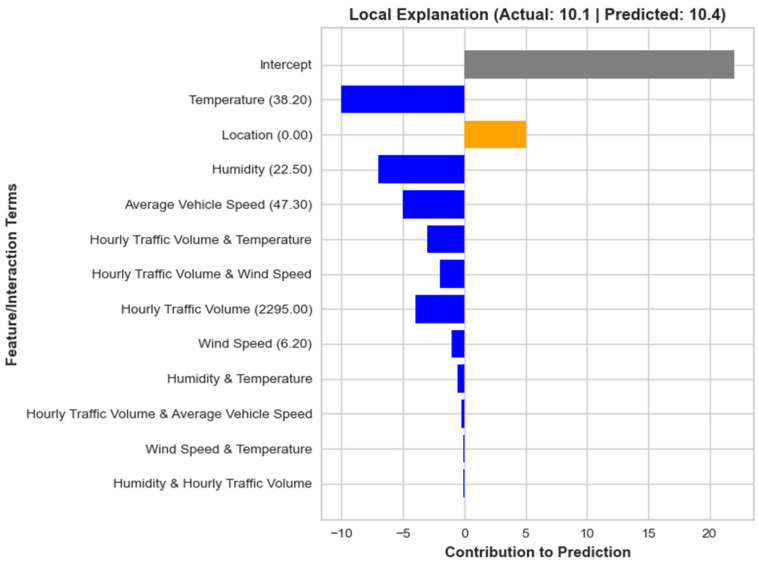
EBM-based local interpretation of Sample # 12 in testing dataset.

This local interpretation plot for sample #38 highlights how the top three factors, temperature (38.20), location (0.00), and humidity (22.50), contribute to the model’s predicted value of 10.4, which is close to the actual value of 10.1, as shown by Figure 12. Temperature (38.20) has the strongest negative influence on the prediction, reducing it by approximately −10 units. This suggests that higher temperatures in this specific case lead to a lower predicted outcome, making temperature one of the key drivers pushing the prediction downward.

In contrast, location (0.00) has a significant positive impact, contributing about five units to the prediction. The specific location of this sample plays a major role in increasing the predicted value, offsetting some of the negative effects from other factors. On the other hand, humidity (22.50) has a substantial negative contribution, lowering the prediction by about −7 units. This indicates that higher humidity levels for this sample are associated with a decrease in the predicted value. Together, these three factors explain much of the predicted outcome, with temperature and humidity reducing the value while location increases it.

## 5. Conclusions and Recommendations

This study developed a Hybrid Explainable Boosting Machine (EBM) framework, optimized with the Covariance Matrix Adaptation Evolution Strategy (CMA-ES), to predict vehicle-related PM_2.5_ concentrations along the Nairobi Expressway. The model effectively captured the influence of environmental and traffic-related factors, providing both high predictive accuracy and interpretability. The findings provide valuable insights for air quality management in developing urban areas:

- The EBM-CMA-ES model was the best-performing model, achieving an MAE of 2.033 and an *R*^2^ of 0.843 on the testing set, significantly outperforming alternative models like XGBoost, RF, and MLR.
- The MLR model performed the worst, with an MAE of 7.226 and an R^2^ of 0.438, indicating its limitations in capturing the complex relationships between environmental factors and PM_2.5_ levels.
- Based on the EBM global interpretation results, location was identified as the most critical factor influencing PM_2.5_ concentrations, with areas near the Westlands roundabout showing the highest levels, likely due to traffic congestion.
- Humidity was found to have a strong positive effect on PM_2.5_ levels, with medium to high humidity linked to increased particle concentrations. Elevated humidity promotes hygroscopic growth, enabling fine particles to absorb water, increasing their size and mass, which elevates PM_2.5_ concentrations. Humidity also enhances aerosol acidity, facilitating secondary aerosol formation. These processes make humidity a critical factor in increasing PM2.5 levels [54]. Temperature showed an inverse relationship with PM_2.5_ concentrations, where higher temperatures were associated with reduced PM_2.5_ levels, likely due to enhanced atmospheric mixing.
- The interaction between humidity and traffic volume was significant, demonstrating that high traffic volume combined with increased humidity results in higher PM_2.5_ concentrations, highlighting the need for targeted interventions in such conditions.

### 5.1. Limitations of Study

This study has several limitations that may affect the generalizability and scope of its findings. Firstly, data were only collected from three monitoring sites along the Nairobi Expressway. This limited spatial coverage may reduce the model’s ability to generalize to other urban regions with varying traffic patterns and environmental conditions. Areas with different road configurations, traffic intensities, or urban layouts may exhibit different PM_2.5_ concentrations, which the current study does not capture.

Moreover, this study considered a limited set of meteorological variables, including humidity, temperature, and wind speed, and did not incorporate other potentially significant factors, such as atmospheric pressure, precipitation, or pollutant interactions. The absence of these variables may lead to an incomplete understanding of the factors influencing PM_2.5_ concentrations. Additionally, the model did not account for temporal dynamics, meaning it did not consider how traffic patterns or weather conditions change over time (e.g., during different seasons or times of the day), which could further affect PM_2.5_ levels.

### 5.2. Future Recommendations

To enhance the accuracy and generalizability of the hybrid EBM-CMA-ES model, future studies should focus on extending data collection over longer periods to capture seasonal and long-term variations in air quality and traffic patterns. By increasing the duration of data collection, the model can be more robust in accounting for temporal dynamics, which are essential for understanding how factors like traffic volume and meteorological conditions fluctuate over time. Additionally, expanding sensor coverage by deploying more air quality monitoring stations along the expressway and in surrounding areas will provide a more comprehensive understanding of PM_2.5_ distribution. This will allow for a finer spatial analysis and improve the model’s ability to generalize to other urban environments.

Incorporating additional meteorological factors, such as atmospheric pressure, precipitation, and solar radiation, will further enhance the model’s predictive capability by accounting for a broader range of environmental influences on PM_2.5_ concentrations. Policy interventions should be targeted in high-risk areas, such as the Westlands roundabout, particularly during periods of high traffic and humidity, when PM_2.5_ levels are likely to peak. Future research should also explore the use of temporal models to account for time-dependent changes in traffic and weather conditions, improving the accuracy of air pollution forecasts and aiding in more informed decision-making for urban air quality management.

## Figures and Tables

**Table 1:** Description and locations of sampling sites in Nairobi.

Sites	Description	Latitude	Longitude
Westlands roundabout (Site 1)	Located on Waiyaki Way, this is a three-lane highway in each direction adjacent to the Westlands roundabout. The area experiences a high proportion of personal vehicles and buses due to its central location and proximity to residential neighborhoods	−1.26551	36.80268
Nyayo roundabout (Site 2)	Situated in Bellevue, this is a three-lane highway in each direction. It is a busy urban route with a balanced mix of personal and commercial vehicles. Although congestion levels here are generally lower than at Westlands, it experiences similar types of traffic.	−1.31940	36.83854
City Cabanas (Site 3)	Positioned near the Airport North Road and Mombasa Road interchange, this site has a three-lane highway in each direction. Given its proximity to the airport and industrial areas, it sees a high volume of heavy vehicles, including goods transport and delivery trucks.	−1.33573	36.89217

**Table 2:** Summary Table of different machine learning models.

Model	Acronym	Key Features	Primary Applications
Extreme Gradient Boosting	XGBoost	− Utilizes gradient boosting with regularization − Highly efficient and optimized for large datasets − Handles missing values automatically	Regression and classification tasks, especially with structured/tabular data
Random Forest	RF	− Ensemble of decision trees with bootstrapping (bagging) − Reduces overfitting by averaging predictions − Robust to noise and outliers	Broad use in classification and regression, especially for feature importance analysis
Light Gradient Boosting Machine	LightGBM	− Optimized for speed and efficiency, handles large datasets − Uses leaf-wise tree growth − Highly effective for sparse data	High-dimensional data in classification and regression, effective with large datasets
Adaptive Boosting	AdaBoost	− Sequentially combines weak learners to form a strong model − Focuses on errors of previous models − Works well with simple base estimators	Binary classification and situations requiring model interpretability

**Table 3:** Summary statistics for different input factors.

Factors	Descriptive Statistics			
	Mean	Standard Deviation	Min	Max
Humidity (%)	37.52	14.95	15.33	70.16
Temperature (°C)	28.75	6.53	18.86	44.07
Average Traffic Volume (veh/hr)	1379.05	655.16	342	3213
Average Vehicle Speed (km/hr)	46.41	9.71	24.7	62.18
Wind Speed (m/s)	6.61	3.83	2.95	11.75
Location	0.94	0.79	0	2

**Table 4:** Hyperparameters of different machine learning models.

Models	Hyperparameters	Range	Optimal Values
EBM	n_estimators	[100, 500]	140
	max_bins	[120, 250]	185
	max_interaction_bins	[30, 120]	70
	learning_rate	[0.01, 0.1]	0.08
XGBoost	learning_rate	[0.01, 0.15]	0.08
	n_estimators	[50, 1000]	600.0
RF	n_estimators	[50, 1000]	420.0
	max_depth	[2, 12]	7.0
LightGBM	learning_rate	[0.01, 0.15]	0.13
	n_estimators	[50, 1000]	800.0
AdaBoost	learning_rate	[0.01, 0.15]	0.06
	n_estimators	[50, 1000]	180.0

**Table 5:** Performance evaluation of EBM, other competitive machine learning models, and a statistical MLR.

Models	Training Dataset				Testing Dataset			
	MAE	MSE	RMSE	*R* ^2^	MAE	MSE	RMSE	*R* ^2^
EBM	1.61	15.53	3.94	0.90	2.03	28.13	5.30	0.84
XGBoost	3.58	31.56	5.62	0.81	3.84	34.58	5.88	0.78
RF	4.26	38.29	6.19	0.77	4.52	40.79	6.39	0.74
LightGBM	4.13	33.68	5.8	0.80	4.27	34.4	5.87	0.78
AdaBoost	7.01	74.39	8.63	0.55	6.75	68.18	8.26	0.57
MLR	7.55	97.98	9.95	0.41	7.23	89.12	9.44	0.43

**Table 6:** Uncertainty analysis in terms of mean ratio and standard deviation of data points.

Models	Mean	Standard Deviation
EBM	1.0024	0.178
XGBoost	0.942	0.195
RF	0.926	0.221
LightGBM	0.934	0.199
AdaBoost	0.786	0.304
MLR	0.772	0.313

## Data Availability

The raw data supporting the conclusions of this article will be made available by the authors upon request.
